# Pre- and postmenopausal women have different core urinary microbiota

**DOI:** 10.1038/s41598-021-81790-8

**Published:** 2021-01-26

**Authors:** Nadia Ammitzbøll, Benedikt Paul Josef Bau, Caspar Bundgaard-Nielsen, Annemarie Brusen Villadsen, Ann-Maria Jensen, Peter Derek Christian Leutscher, Karin Glavind, Søren Hagstrøm, Louise Thomsen Schmidt Arenholt, Suzette Sørensen

**Affiliations:** 1 Centre for Clinical Research, North Denmark Regional Hospital, Bispensgade 37, 9800 Hjørring, Denmark; 2 Department of Clinical Medicine, Aalborg University, Aalborg, Denmark; 3 Department of Gynecology and Obstetrics, North Denmark Regional Hospital, Hjørring, Denmark; 4 Department of Obstetrics and Gynecology, Aalborg University Hospital, Aalborg, Denmark; 5 Department of Pediatrics, Aalborg University Hospital, Aalborg, Denmark

**Keywords:** Microbiology, Bladder

## Abstract

Recent studies suggest that alterations in the female urinary microbiota is associated to development of bladder disease. However, the normal microbiota composition and variation in healthy women are poorly described. Moreover, the effects of hormonal changes on microbiota during menopause is not well understood. The aim of our study was to investigate the urinary microbiota in healthy pre- and postmenopausal women without urinary tract symptoms. Microbiota composition in catheterized urine samples was mapped using 16S rRNA gene sequencing. In total, 41 premenopausal and 42 postmenopausal women were initially included. Samples with first PCR amplification concentration below level of the negative control were excluded, resulting in 34 premenopausal and 20 postmenopausal women included in data analysis. Urine from postmenopausal women showed significantly higher alpha diversity compared to premenopausal women. *Lactobacillus* was the most abundant bacteria in both groups, however the relative abundance of *Lactobacillus* accounted for 77.8% in premenopausal versus 42.0% in postmenopausal women. In conclusion, urine from premenopausal mostly presented with *Lactobacillus* dominated urotypes*,* whereas urine from postmenopausal women presented a more diverse urinary microbiota with higher abundance of the genera *Gardnerella* and *Prevotella*. The clinical and pathophysiological implications of this difference remain to be elucidated.

## Introduction

The human urinary microbiota has recently received much attention in connection to bladder health and function. Based on standard culturing methods, urine from healthy bladders has traditionally been considered sterile. Application of more sensitive and specific techniques, such as next generation sequencing and enhanced urine culturing, has revealed the presence of a naturally dwelling bacterial community in urine from healthy individuals without urinary tract symptoms^1–4^. Previous studies have reported that the female urinary microbiota can be divided into different urotypes, depending on their dominant bacterial genera. Some disagreement exists regarding the number of urotypes, depending on collection and analysis methods^5–9^. However, the studies agree that the majority of women have a urotype dominated by *Lactobacillus*, together with other common bacteria, including *Gardnerella, Prevotella,* and *Streptococcus*^1,2,9,10^. The function and implications of the urinary microbiota is largely unexplored. However, the first studies are emerging, pointing towards an association between a dysbiotic urinary microbiota and bladder disorders such as urgency urinary incontinence (UUI)^5,11–13^, overactive bladder (OAB)^14^, neuropathic bladder^15^, and recurrent urinary tract infections (rUTI)^16^. Whether there is a causal relationship between this altered microbiota and urinary tract disease remains unknown.

UUI, OAB, and rUTIs are common in women of all ages, with an increased risk after menopause^17–19^. The underlying etiology is unclear, however, a plausible explanation may be found in possible age- or hormonal status dependent differences in the urinary microbiota^9,20–22^. Microbiota alterations in relation to age and/or hormone changes during menopause have been observed in other microbial niches such as the gut and vaginal microbiota^23,24^. In the vaginal microbiota, a study reported that the bacterial composition was dependent on menopausal status, in which *Lactobacillus* was more dominant in pre- and perimenopausal women compared to postmenopausal women^24^. Additionally, local vaginal estrogen treatment in postmenopausal women have shown increased level of *Lactobacillus* in the vagina^25^ and the bladder^26^. Both local vaginal estrogen treatment and treatment with *Lactobacillus* supplements in patients suffering from rUTIs reduces the risk of infections^25,27,28^. Together, this indicate a possible role of estrogen level on the urinary microbiota composition. Therefore, we aimed at investigating the female urinary microbiota in healthy pre- and postmenopausal women to study if the urinary microbiota of the bladder varies with age and/or menopausal status.

## Results

In this study, 87 women were included of which 43 were premenopausal women and 44 postmenopausal women. Four women were subsequently excluded based on a positive standard urine culturing, leaving 41 premenopausal (mean age 37 ± 9.5 years) and 42 postmenopausal (mean age 63 ± 6.9 years) women in the final study. All women were ethnic Danes. Characteristics such as BMI and smoking status were comparable between the two groups (Table 1). The postmenopausal women had a higher mean number of total births but less caesarean sections compared to premenopausal women.

**Table 1:** Demographic characterization of study participants.

Participants n = 83	Premenopausal n = 41 (49.4%)	Postmenopausal n = 42 (50.6%)	
**Age**			
Range	18 – 49	55 – 81	
Mean (Years (± SD))	37.2 (± 9.5)	63.3 (± 6.9)	*p* = 4.35 × 10^−15^
**BMI**			
Mean (kg/m^2^ (± SD))	26.4 (± 5.2)	25.8 (± 4.4)	*p* = 0.067
**Smoking status (n (%))**			
Never	19 (46.3%)	29 (69.0%)	*p* = 0.095
Previously	17 (41.5%)	11 (26.2%)	*p* = 0.095
Currently	5 (12.2%)	2 (4.8%)	*p* = 0.095
**Total births (n(± SD))**			
Mean births	1.6 (± 1.2 SD)	2.5 (± 1.0 SD)	*p* = 0.002
Mean caesarean section	0.4 (± 0.7 SD)	0.2 (± 0.7 SD)	*p* = 0.039

DNA purification from the urine samples showed presence of DNA, albeit for some samples at low concentrations, ranging from below 1 ng/mL urine to 15,000 ng/mL urine. To avoid miss- or over-interpretation of bacterial status, the DNA concentration was subsequently evaluated after the initial PCR during library preparation for 16S ribosomal RNA (rRNA) gene sequencing. Samples that produced lower DNA concentrations following library generation, compared to the negative controls, were excluded in all subsequent analyses (supplementary Fig. S1a). This resulted in urine samples from 29 women being excluded, leaving 34 women in the premenopausal group and 20 women in the postmenopausal group in the data analysis. Subgroup analyses revealed that the excluded women were older (*p* = 0.021), contained fewer active smokers (*p* = 0.036 and 0.030 for all women and postmenopausal women only, respectively), and that there was a higher proportion of postmenopausal women among the excluded women compared to the included women (*p* = 0.017). No differences were observed in BMI and age after accounting for the variation in number of premenopausal and postmenopausal women (supplementary Fig. S2). After removal of these samples, quality filtering, and chimera removal, a total of 2,467,401 reads were obtained with a mean number of 24,674.01 reads per sample. A total of 2,449 unique Operational Taxonomic Units (OTUs) were identified, with 93.14% being identified at the phylum taxonomic level, 50.84% at the genus level, and 0.08% at the species level. A rarefaction curve showed good sequencing coverage (supplementary Fig. S1b).

### The urinary microbiota in premenopausal women is different from that of postmenopausal women

**Figure 1:**
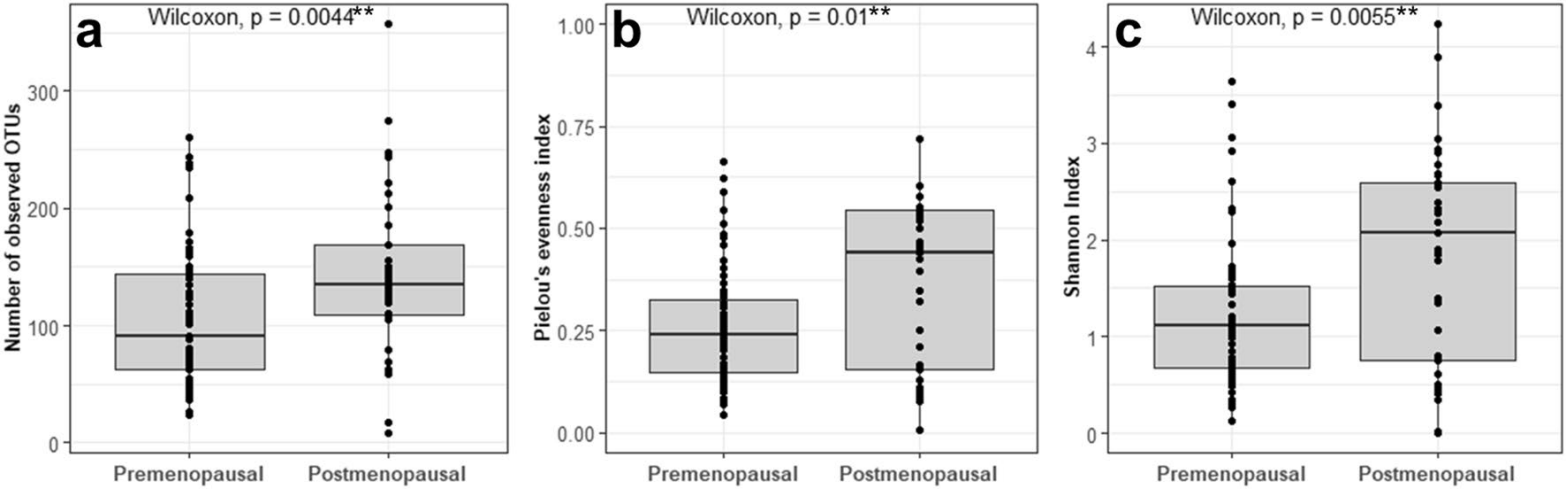
Alpha-diversity of the urinary microbiota in pre- and postmenopausal women depicted using (**a**) Number of observed OTUs, (**b**) Pielou’s evenness index, and (**c**) Shannon diversity index.

**Figure 2:**
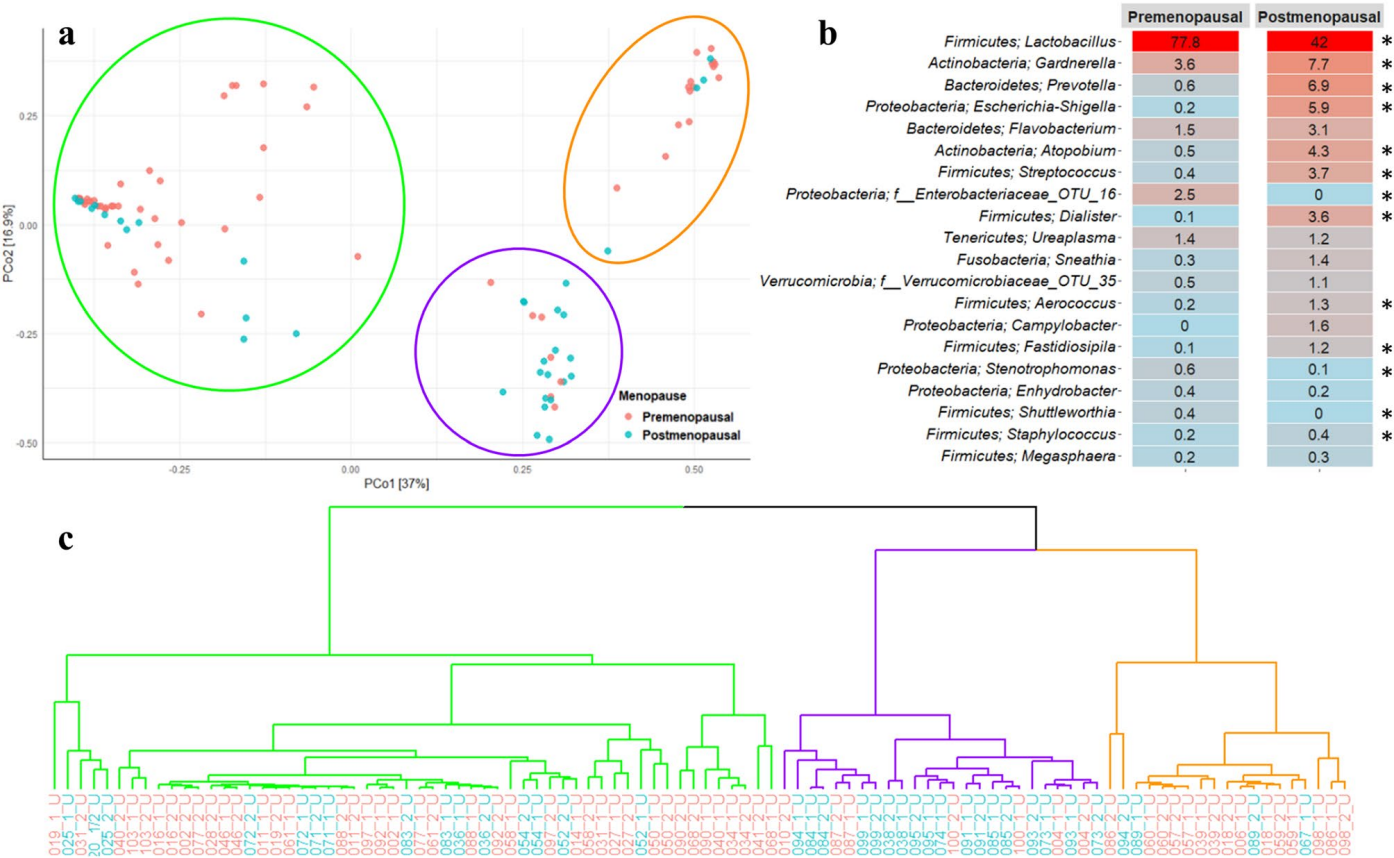
The bacterial composition in urine samples from pre- and postmenopausal women. (**a**) PCoA plot using Bray Curtis dissimilarity. Three urotypes were identified, as indicated with green, purple and orange circles. (**b**) Heatmap, indicating the 20 most abundant bacterial genera in the urine, as well as percentage of total OTUs. Red and blue respectively indicates high and low relative abundance. *Benjamin Hochberg adjusted *p* value < 0.01. (**c**) Dendrogram obtained with hierarchical clustering of OTU diversity in samples, based on the PCoA plot. Branch colors represent urotype, while label color represent menopausal state: red indicate premenopausal women, whereas blue represent postmenopausal women.

When comparing the bacterial composition in urine from premenopausal women with that of postmenopausal women, we observed a statistically significant difference in alpha diversity. A significantly lower OTU richness (*p* = 0.004), Pielou evenness (*p* = 0.010), and Shannon diversity index (*p* = 0.005) was observed in the urine of premenopausal women compared to that of postmenopausal women (Fig. 1a–c). Beta diversity was analyzed, using Principal Coordinates Analysis (PCoA), showing a segregation of pre- and postmenopausal women (Fig. 2a). We investigated the resulting clusters using hierarchical cluster analysis and observed that the urinary microbiota separated into three distinct urotypes. Two of the urotypes were predominantly found amongst premenopausal women, whereas the last urotype was mostly observed amongst postmenopausal women (Fig. 2c). Heatmap representation of the 20 most abundant genera showed that urine of both the pre- and postmenopausal women were dominated by the *Lactobacillus* genus (Fig. 2b), however, the relative abundance of these were markedly lower in the postmenopausal women (42.0%) compared to the premenopausal women (77.8%, Benjamin-Hochberg corrected *p* value < 0.01). Instead, the postmenopausal women appeared to have a broader representation of different bacteria, as supported by the higher alpha diversity, including higher relative abundances of *Gardnerella*, *Prevotella*, *Escherichia-Shigella*, *Atopobium*, *Streptococcus*, and *Dialister*.

Since we observed different urotypes in the two groups, we analyzed pre- and postmenopausal women separately, to determine if variations in core microbiota existed within the groups.

**Figure 3:**
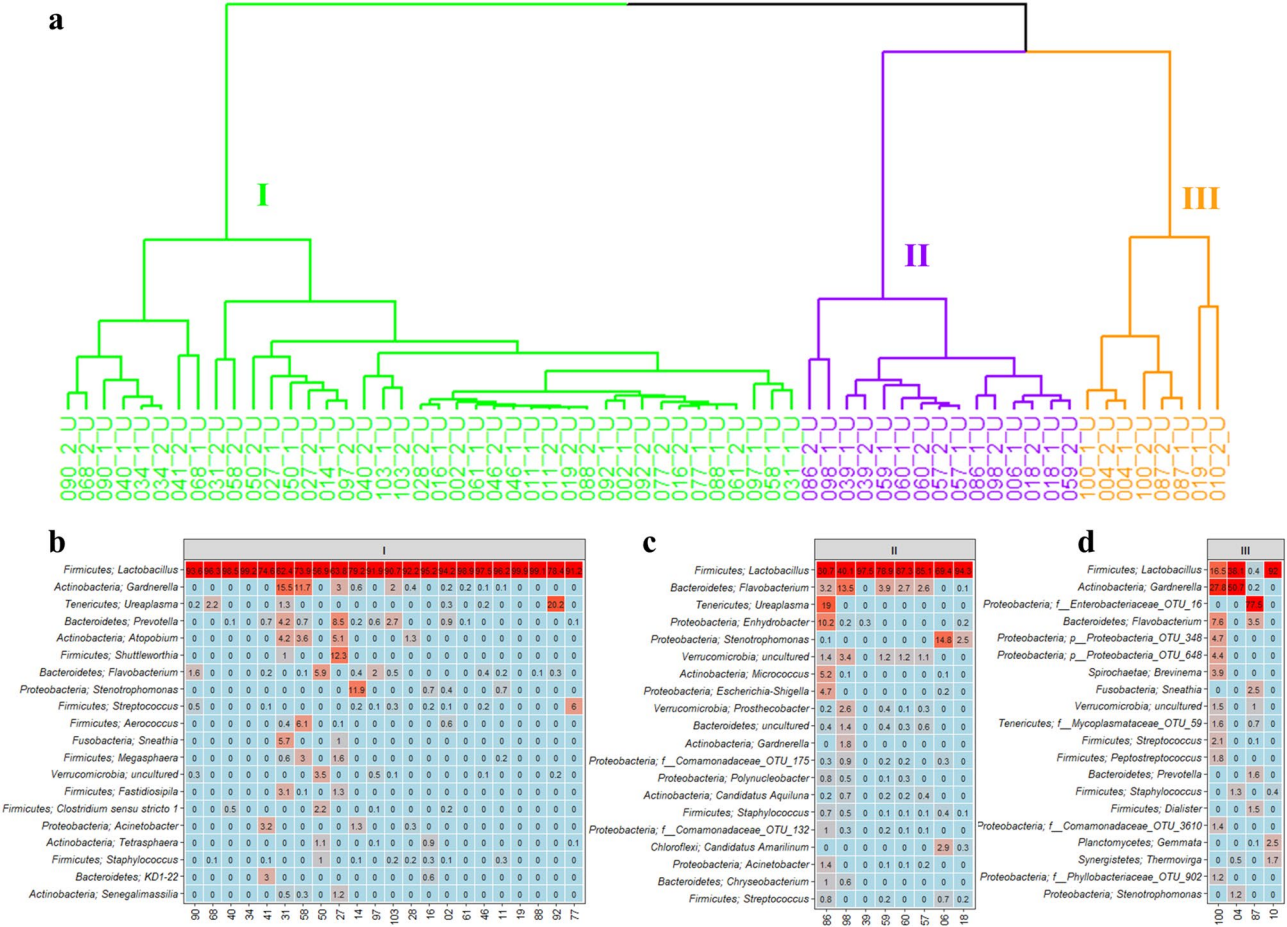
The bacterial composition in urine samples from premenopausal women. (**a**) Dendrogram obtained with hierarchical clustering of OTU diversity in samples. Three urotypes were identified, with green, purple and orange branches representing urotype I, II, and III respectively. (**b**–**d**) Heatmaps showing the 20 most abundant bacterial genera in the three urotypes, I, II and III. The premenopausal donors 2, 4, and 6 were not placed in any clusters by the unsupervised clustering algorithm used.

Hierarchical cluster analysis demonstrated that the bacterial composition of the premenopausal women separated into three urotypes (Fig. 3a). In urotype I, representing the majority of premenopausal women, *Lactobacillus* was the primary genus accounting for a high relative abundance with a small contribution from *Gardnerella* (Fig. 3b). The second urotype, urotype II, was likewise dominated by *Lactobacillus* (Fig. 3c) with secondary contributions from *Flavobacterium*, *Ureaplasma*, and *Enhydrobacter*. In addition to these *Lactobacillus*-dominated urotypes, a third urotype, urotype III, appeared with a more mixed bacterial distribution (Fig. 3d).

**Figure 4:**
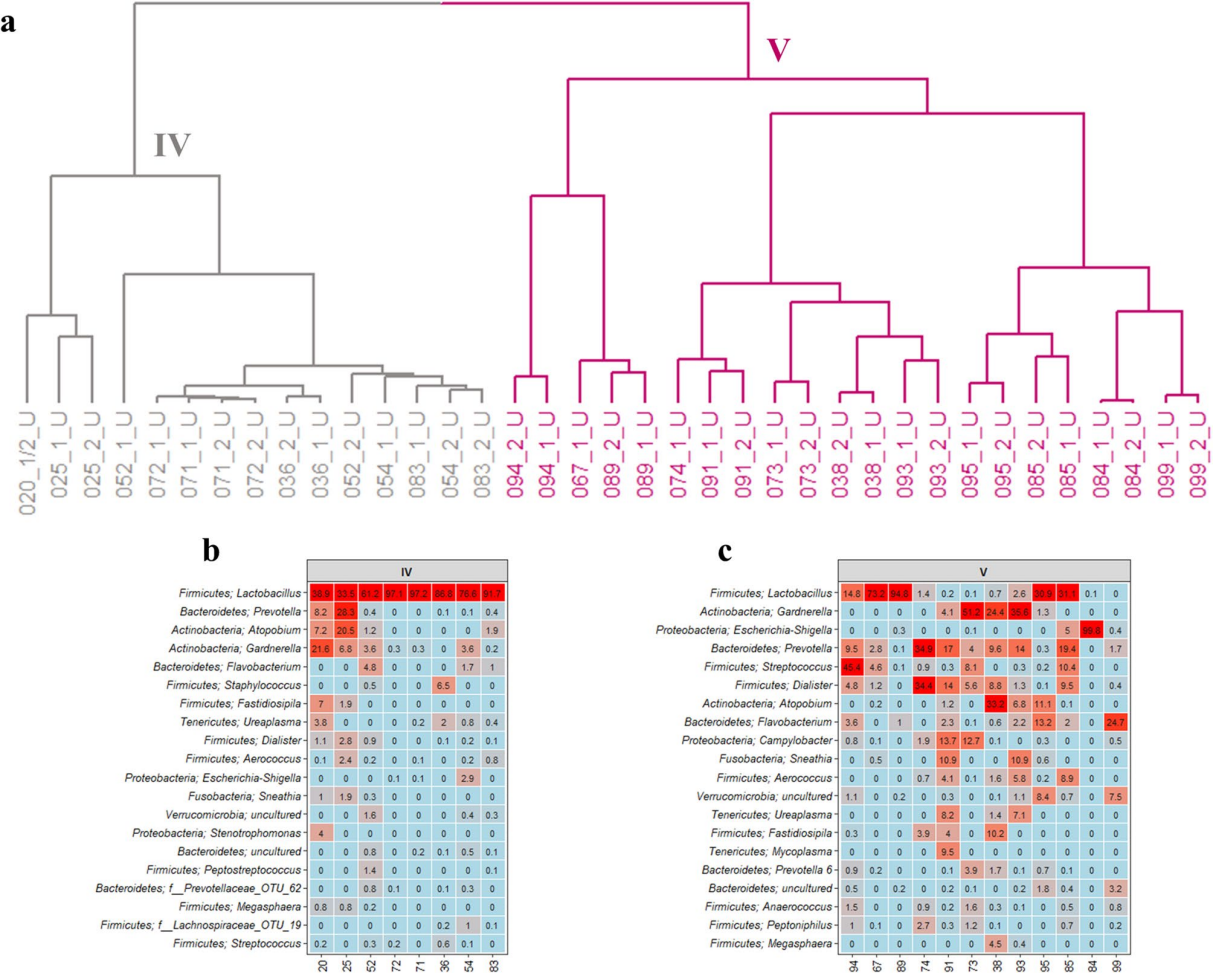
The bacterial composition in urine samples from postmenopausal women. (**a**) Dendrogram obtained with hierarchical clustering of OTU diversity in samples. Two urotypes were identified, with grey and mangenta colored branches representing urotype IV and V respectively. (**b**,**c**) Heatmaps showing the 20 most abundant bacterial genera in the two urotypes.

The postmenopausal women were similarly grouped into two separate urotypes (Fig. 4a). The first urotype of postmenopausal women, urotype IV, showed high similarity to urotype I and II for premenopausal women by being highly dominated by *Lactobacillus* (Fig. 4b). In contrast, the second urotype, urotype V, was defined by a highly diverse bacterial composition with less abundant *Lactobacillus* and higher contributions from *Gardnerella*, E*scherichia-Shigella*, *Prevotella*, *Streptococcus*, *Dialister*, *Atopobium*, and *Flavobacterium* (Fig. 4c).

**Figure 5:**
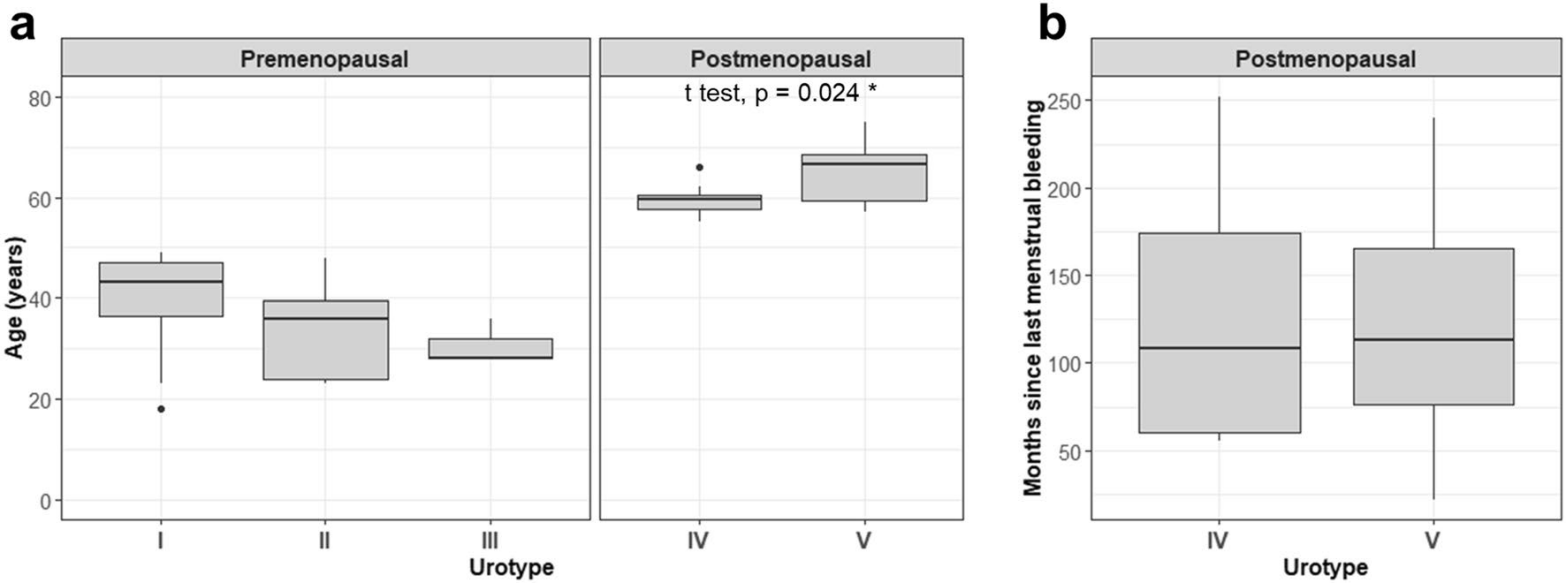
Demographics of the urotypes from pre- and postmenopausal women. (**a**) Age of the different urotypes in pre- and postmenopausal women. (**b**) Months since last menstruation for postmenopausal women. * *p* = 0.041.

To evaluate whether differences in age, and thereby proximity to menopause, could explain what urotype defined the women, we compared the mean ages of the participants in each urotype. There was no significant difference in the mean age between premenopausal women with urotype I, II, or III (Fig. 5a), indicating that the low-*Lactobacillus* profile observed in urotype III was not associated with age. Conversely, the two urotypes observed amongst the postmenopausal women appeared to be defined by age (*p* = 0.041, Fig. 5a) but not by time since last menstruation (Fig. 5b). This indicates that after menopause, the age of the women may influence the composition of the urinary microbiota.

## Discussion

The composition and normal variation in the urinary microbiota throughout life in healthy women is poorly described, and the effects of hormonal changes, especially during menopause, is not well understood. Our study showed that pre- and postmenopausal women have different core urinary microbiotas, with a predominance of *Lactobacillus* in both groups, but a lower abundance of *Lactobacillus* together with more mixed composition in postmenopausal women. Our results are partly in line with previous studies detecting the bacterial population in healthy females, showing that the urinary microbiota of healthy women is dominated by a few genera, including *Lactobacillus*, *Gardnerella*, and *Prevotella*^3,5,10,11,15,28^. These studies, however, vary in age criteria for study participants and often describe a mixed representation of women ranging from young premenopausal women to late postmenopausal women. Similar to our study, Price et al.^9^ compared the urinary microbiota from catheterized samples between pre- and postmenopausal women. They reported *Lactobacillus* to be the most common urotype (defined by > 50% relative abundance), however, it appeared that this was not associated with age or menopausal status. The reason for this discrepancy between their and our results are unknown, but it may be explained by differences in age or ethnicity. Moreover, methodical differences may also explain some of the differences, as Price et al.^9^ included enhanced bacteria culture together with their 16S rRNA gene sequencing results. In contrast, we only used 16S rRNA gene sequencing and some bacteria may therefore be differentially detected. As an example, in the culture data from Price et al.^9^ they found a high number (9.4%; defined by > 50% relative abundance) of women with a *Streptococcus* dominated urotype, whereas we only identified one woman (1.2%) in our study, with > 40% relative abundance of *Streptococcus*. However, similar to our results, the 16S rRNA gene sequencing data from Price et al.^9^ reported lower relative abundance of *Streptococcus*, indicating that methodically differences highly impact the results.

It is well known that low alpha diversity, with predominant *Lactobacillus* colonization of the vaginal flora is imperative for vaginal health^29,30^ and that a reduction in *Lactobacillus* abundance may cause vaginal disease^30^. Since the vaginal and urinary microbiotas appear to have functional and structural overlaps^31^ it is reasonable to presume that a urotype with low relative abundance of *Lactobacillus* in urine, may be an indicator of bladder dysbiosis and therefore constitutes an increased risk of developing bladder disorders. In our study, the majority of the premenopausal women belonged to one of two urotypes characterized by high relative abundance of *Lactobacillus;* however, we observed a small subgroup of participants belonging to a more mixed urotype, with a lower relative abundance of *Lactobacillus*. This could represent a dysbiotic urinary microbiota putting these women in higher risk of experiencing future bladder related diseases. In line with this, younger women with a low or moderate-*Lactobacillus* dominated urotype have been found to be associated with mixed urinary incontinence, pointing towards a dysbiosis in women lacking *Lactobacillus*^32^. Conversely, it might represent personal variations in the urinary microbiota or be influenced by temporal factors that could change the microbial composition, such as menstruation or coitus^33^.

The menopausal reduction of estrogen has been shown to cause a decrease in the level of free glycogen in the vaginal epithelium in postmenopausal women^34^. As glycogen serves as nutrition for the *Lactobacillus*, a reduced level of *Lactobacillus* in the vagina is observed^34,35^. Therefore, transition to a less *Lactobacillus* dominated urotype in postmenopausal women might be a result of decreasing levels of estrogen^24^. In our study, the postmenopausal women with the *Lactobacillus* dominated urotype, urotype IV, were significantly younger than the women in urotype V. However, this was not related to time since last menstruation, and it is thus unclear if the bacterial variations was related to fluctuations in hormonal levels. Studies investigating the direct role of estrogen on the urinary microbiota are thus urgently needed, in order to evaluate the role of hormonal changes on the bacterial composition in the bladder.

UUI, OAB and other lower urinary tract symptoms are common in elderly women affecting around 12–55%^18^ and the cause behind this may be the altered urinary microbiota following menopause^36^. When looking at the vaginal microbiota, women with lack of *Lactobacillus* spp., especially the loss of peroxide-producing *Lactobacilli*, have increased risk of genital disease, including bacterial vaginosis, increased risk of susceptibility of sexually transmitted infections, pre-term delivery, miscarriage, and pelvis inflammatory disease^37,38^. If the same is true for the urinary microbiota, decrease in *Lactobacillus* abundance could make the women more prone to urinary tract disorders. Previous studies^5,6,39^ have reported an altered bacterial composition in women suffering from UUI, characterized by a more diverse and rich bacterial composition, higher abundance of *Gardnerella,* and significantly decreased *Lactobacillus* compared to healthy controls. These results match our findings in the postmenopausal group. Neither Pearce et al.^5^ nor Thomas-White et al.^6^ used age- and menopausal status matched controls for the two groups (women with UUI being significantly older), and the observed variations may therefore reflect age differences rather than disease state. Furthermore, Price et al.^39^ utilized expanded quantitative urine culture to asses bacterial abundance, which has been demonstrated to result in different bacterial compositions of urine compared to 16S rRNA gene sequencing^9^. Intriguingly, the urinary microbiota profile of the postmenopausal women in our study bear high resemblance to what has been reported for the women with UUI^5,6^. Notably, in a study by Karstens et al.^11^ using age- and menopausal matched controls, they did not find any difference in alpha diversity or in relative abundances of *Gardnerella* and *Lactobacillus* between UUI patients and controls. Larger studies, however, on UUI are needed to conclude on a role of the urinary microbiota in UUI disease. Our study demonstrates that age and menopausal status should be considered in future studies on urinary microbiota and that pre- and postmenopausal women cannot be compared to each other due to significant different microbiota composition.

A number of limitations in our study need to be addressed. First, four asymptomatic women were excluded based on a positive standard urine culture. Uropathogens, specially *E.coli*, has however been found above the typical threshold in asymptomatic women in the study by Price et al.^40^, indicating that exclusion of these four women could have impacted our results. In future studies, one should include women with a positive standard urine culture if they have no clinical symptoms of urinary tract infection. Second, the use of 16S rRNA gene sequencing only allowed us to reliably classify bacteria at phylum and genus level, and specific variation at species level was therefore not evaluated. Studies have previously implicated *Lactobacillus* species, like *L. gasseri* and *L. iners*, to play a function in bladder diseases such as UUI and neuropathic bladder^5,41^, indicating that different *Lactobacillus* species may have different functional effects on bladder health. Third, use of 16S rRNA gene sequencing and enhanced bacteria culture may give different urinary microbiota results^9^ and one may argue that both methods should be included to proper reflect the microbiota of the bladder. Fourth, we only collected urine at a single timepoint, and as the bacterial composition has been shown by some to fluctuate over time^33^, this possible lack of temporal stability may have impacted the results. We have, however, previously shown that the composition of the urinary microbiota remains stable over a shorter time period^42^. Finally, we did not account for other factors that could potentially influence the urinary microbiota, such as diet^43^, fluid intake, urinary oxygen concentrations^44^, menstruation cycle^33^, sexual activity^33^, and vulvo-vaginal symptoms^45,46^. This could in particular be a limitation since these would be expected to differ highly between our two groups.

However, this study also has strengths. This is one of the first studies investigating a difference between pre- and postmenopausal women using catheterized urine samples instead of midstream urine samples, minimizing vulva-vaginal contamination^1,47^ and thereby likely giving a more representative picture of the urinary microbiota. For instance, a relatively high number of samples were excluded due to DNA concentrations being below negative control cut-off levels (after the initial PCR reaction in library preparation). This is consistent with other studies investigating urine obtained by transurethral catheterization^1,5,6,12^, and may not be a technical issue, but rather reflect a biologically relevant variation in urinary bacterial content. Importantly, the excluded women were older and less likely to be smokers, which corroborate this theory. Studies using voided urine, in contrast, obtained a higher bacterial DNA yield^2,48,49^, suggesting contamination with urethral bacteria. Moreover, in our statistical analyses possible confounders including smoking status, age, and BMI have been taken into account, increasing the strength of the study. Additionally, we included duplicates of our urine samples increasing the reproducibility of our results. Finally, the characterization of study participants regarding in- and exclusion criteria is highly well defined; e.g. by the use of two validated symptom questionnaires.

## Conclusion

This study demonstrated that pre- and postmenopausal women overall possess different urinary microbiotas. Urine from women prior to menopause was dominated by *Lactobacillus* whereas postmenopausal women tended to display a more diverse urinary microbiota. Moreover, in healthy women, a normal variation in urinary microbiota might be present and a shift in urinary microbiota may occur during menopause that need to be emphasized when investigating dysbiosis of the bladder. Results from our study extend the knowledge of the normal microbiota in women and the role of age and/or menopausal stage on the microbial composition.

## Methods

### Study participants and clinical data

Women were recruited by advertisement on social medias and in the newspaper. The study was approved by The North Denmark Region Committee on Health Research Ethics (N-20170050) and all women provided informed consent. The investigation was conducted according to the principles expressed in the Declaration of Helsinki. The study was registered at the Danish Data Protection Agency.

The premenopausal group consisted of non-pregnant women aged 18–50 years, while postmenopausal women were aged > 55 years and without vaginal bleedings within one year of study inclusion. Exclusion criteria for both groups included bladder symptoms, antibiotic treatment within three months prior to inclusion, recurrent cystitis (> 2 per year) or current UTI evaluated by a positive urine culture of the collected urine sample. For the postmenopausal group, use of hormonal replacement therapy (oral and topical) was considered an exclusion criterion. Clinical assessment of bladder symptoms was evaluated by two questionnaires designed to measure frequency and severity of urinary incontinence; the International Consultation on Incontinence Questionnaire (ICIQ) Urinary Incontinence Short Form (ICIQ-UI-SF)^50^ and Overactive Bladder (ICIQ-OAB)^51^. Additional data was collected, including menstrual status, use of hormonal contraceptives, BMI, age, caesarean section, and previous births.

### Urine collection

All samples were collected at the Department of Obstetrics and Gynecology, North Denmark Regional Hospital, using a CH/FR 12/4.0 mm urinary catheter (SpeediCath, Coloplast Denmark) for sterile intermittent catheterization. Prior to catheter insertion, the urethral meatus was cleaned with sterile water. Urine was collected in a 50 mL collection tube, aliquoted into 10 mL fractions within 15 min, and immediately placed at -80 °C until further processing. Ten mL of urine was collected in a Urine Monovette with boric acid (Saarstedt, Germany) for standard urine culture at the Department of Clinical Microbiology, Aalborg University Hospital.

### DNA extraction

From 10 mL of urine, bacterial DNA was isolated using the QIAamp Viral RNA Mini Kit (Qiagen) according to manufacturer´s recommendations, modified with a pre-treatment step, as previously explained^42^. All samples were extracted and analyzed as duplicates. Briefly, before DNA extraction, urine samples were centrifuged at 3,220 × g for 20 min. Pellets were resuspended in lysis buffer and a bead beating step was included using the TissueLyser LT (Qiagen) for 2 min at 30 Hz with a 5 mm stainless steel bead. DNA yield was measured by fluorometric quantification using the Qubit 4 Fluorometer (Thermo Fisher) with the Qubit dsDNA Broad Range Assay Kit (Thermo Fisher). Reagent and procedure contamination controls were included by performing the DNA extraction on Nuclease-free water.

### 16S rRNA Gene Sequencing and bioinformatics

Bacterial composition of urine samples were evaluated by 16S rRNA gene sequencing, targeting the V4 hypervariable region, on the Illumina MiSeq platform. Library preparation and sequencing was performed by DNASense (Denmark), as previously described^52^, using ≤ 5 ng/µL DNA for library preparation. For error rate estimation during sequencing, a 20% PhiX control library (Illumina) was used. A second negative control, consisting of nuclease-free water, was sequenced to eliminate background contaminants. Finally, a positive control (complex bacteria sample obtained from an anaerobic digester system) was used to monitor sequencing efficiency and minimize batch effects.

Forward reads were trimmed for quality using Trimmomatic v. 0.32^53^ utilizing settings: SLIDINGWINDOW:5:3 and MINLEN:250 to remove poor quality reads and discard reads < 250 bp in length. Reads were dereplicated and processed using the UPARSE pipeline^54^. Dereplicated reads were clustered using the usearch v. 7.0.1090 command “-cluster_OTUs” with default settings, and OTUs were formed based on 97% identity, using the “-usearch_global”. Finally, bacterial taxonomy was assigned using the RDP classifier^55^, with the command “parallel_assign_taxonomy_RDP.py” in QIIME^56^ and the MiDAS database v.1.20^57^.

### Statistics

Data analysis was performed using R version 3.6.1^58^ through the Rstudio IDE (http://www.rstudio.com/). 16S rRNA data was analyzed using the ampvis2 package v.2.4.12^59^. Alpha diversity was determined using OTU richness and Shannon diversity index, whereas β diversity was determined using PCoA based on Bray–Curtis distance measures. To further analyze differences and similarities, we performed unsupervised hierarchical cluster analysis on the Euclidian distance between OTUs, and visualized the results with dendrograms, using the dendextend package v. 1.13.2^60^. This dendrogram was furthermore used to classify urotypes. To determine OTUs with statistically significant distributions in pre- and postmenopausal women, we used the DESeq2 package v.1.24.0^61^ to generate multiple hypothesis corrected *p* values using the Benjamini–Hochberg procedure^62^.

For continuous data, like OTU richness, Shannon diversity index, age etc., distribution and variance were assessed using Shapiro-Wilks test and Bartlett’s test, respectively. Normal distributed data with equal variance were compared using Student´s t-test or ANOVA followed by Tukeys post hoc test. Non-parametric data were compared using Mann–Whitney-Wilcoxon test or Kruskall-Wallis test followed by Dunn’s post hoc test. A *p* value of < 0.05 was considered significant for all statistic tests, whereas for multiple hypothesis corrected *p* values, a padj of < 0.01 was required.

## Supplementary Information

Supplementary Information 1.

Supplementary Information 2.

Supplementary Information 3.

## Data Availability

All data generated or analyzed in the study, are included in this published article and its supplementary information files. Sample information (supplementary_metadata) and OTU-tables (Supplementary_Otutable) generated during sequencing and used for bioinformatics are included as supplementary files.
