# Supplementary material for: Pre- and postmenopausal women have different core urinary microbiota
Source: Sci Rep. 2021 Jan 26;11:2212. doi: 10.1038/s41598-021-81790-8 (PMC7838182; doi:10.1038/s41598-021-81790-8)
Supplement: Supplementary file 1 — Supplementary Information 1. [file 41598_2021_81790_MOESM1_ESM.pdf]

Pre- and postmenopausal women have different core urinary microbiota

Nadia Ammitzbøll, Benedikt Paul Josef Bau, Caspar Bundgaard-Nielsen, Annemarie Brusen Villadsen, Ann-Maria Jensen, Peter Leutscher, Karin Glavind, Søren Hagstrøm, Louise Thomsen Schmidt Arenholt, Suzette Sørensen

Supplementary Figure S1. Sequencing quality

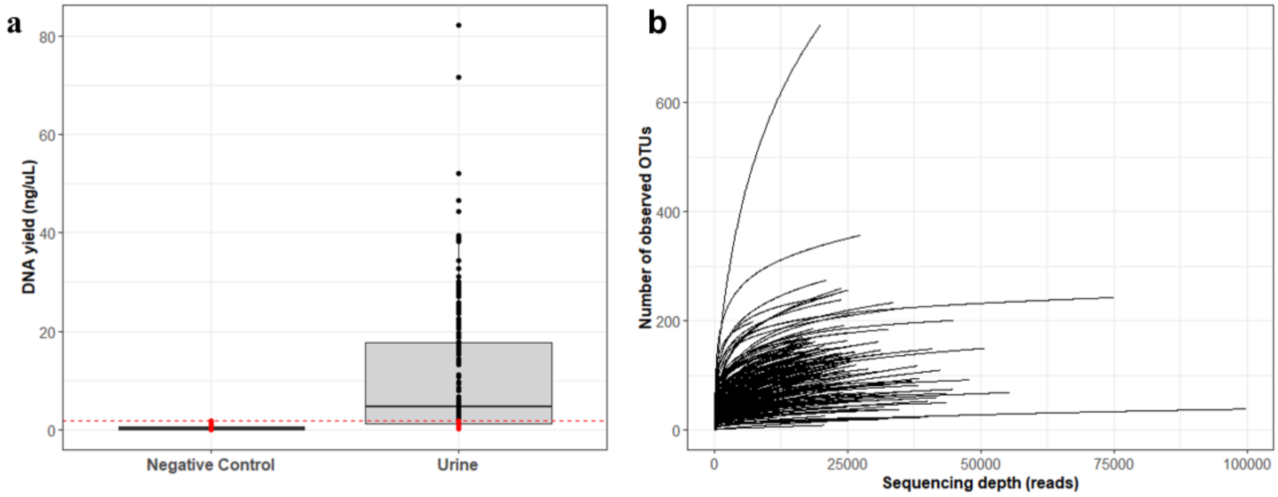

Coverage of sequencing run. a) The DNA concentration after library generation using primers targeting the V4 region of the 16S rRNA gene. The red line illustrates the cut-off value at 1.84 ng/µl. All experiments were performed in duplicates. b) Rarefaction curve visualizing coverage of reads in all samples.

Supplementary Figure S2. Comparison of excluded and included women

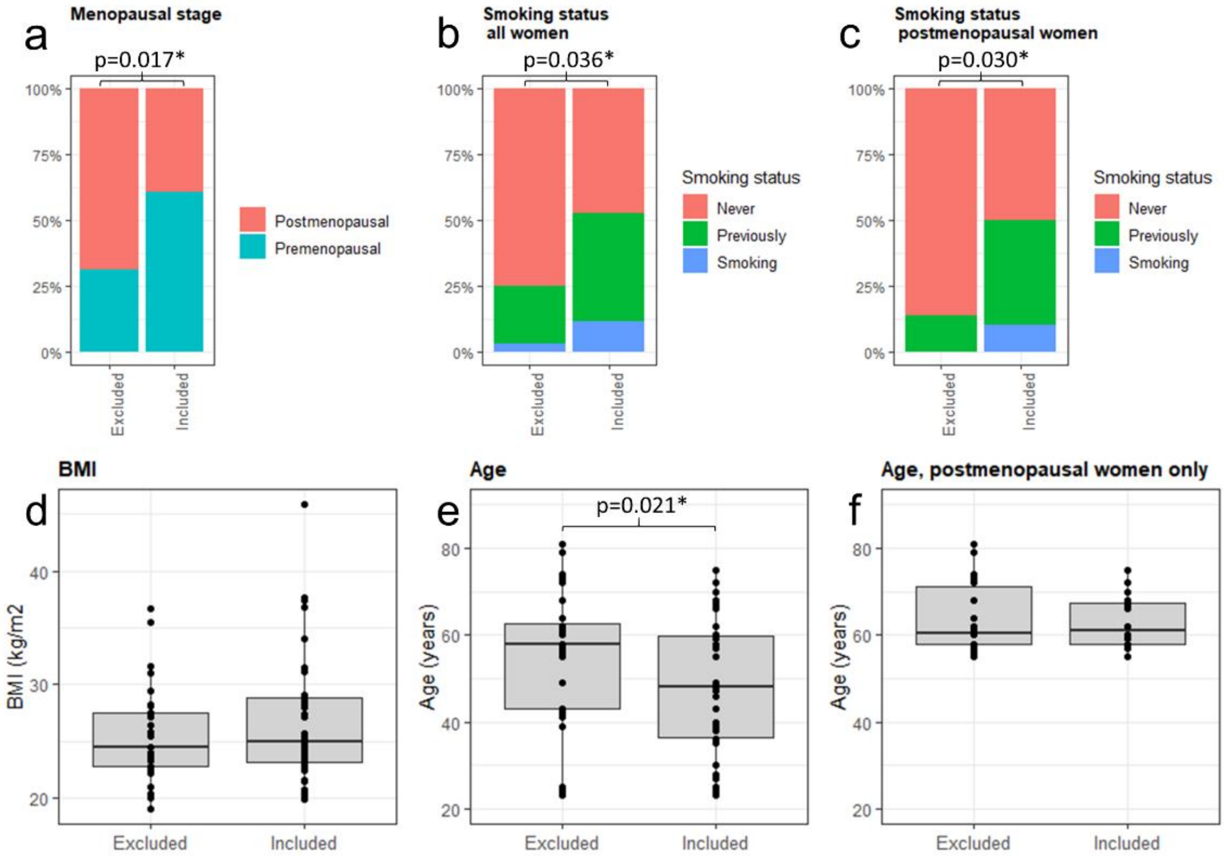

Comparison of demographics between women, whose samples produced too few reads and thus were excluded as described in supplementary figure 1a ("excluded", 29 women), compared to women with sequencing results ("included", 54 women). a) Proportion of pre- and postmenopausal women amongst the excluded and included women. b) and c) Smoking status of all women (b) and postmenopausal women (c), defined as current smokers, previous smokers or women that have never been smokers. d) BMI of included and excluded women. e) and f) Comparison of age between excluded and included women, separated for pre- and postmenopausal women combined (e) and postmenopausal women alone (f). Statistics are calculated using Chi2 test (a-c), and two sample Mann-Whitney U test (d-f).
